# Interfacial photochemistry at the ocean surface is a global source of organic vapors and aerosols

**DOI:** 10.1038/s41467-018-04528-7

**Published:** 2018-05-29

**Authors:** Martin Brüggemann, Nathalie Hayeck, Christian George

**Affiliations:** 1 Univ Lyon, Université Claude Bernard Lyon 1, CNRS, IRCELYON, F-69626 Villeurbanne, France; 2 Present Address: Leibniz Institute for Tropospheric Research (TROPOS), Permoserstr. 15, 04318 Leipzig, Germany

## Abstract

The surface of the oceans acts as a global sink and source for trace gases and aerosol particles. Recent studies suggest that photochemical reactions at this air/water interface produce organic vapors, enhancing particle formation in the atmosphere. However, current model calculations neglect this abiotic source of reactive compounds and account only for biological emissions. Here we show that interfacial photochemistry serves as a major abiotic source of volatile organic compounds (VOCs) on a global scale, capable to compete with emissions from marine biology. Our results indicate global emissions of 23.2–91.9 TgC yr^–1^ of organic vapors from the oceans into the marine atmosphere and a potential contribution to organic aerosol mass of more than 60% over the remote ocean. Moreover, we provide global distributions of VOC formation potentials, which can be used as simple tools for field studies to estimate photochemical VOC emissions depending on location and season.

## Abstract (web summary)

Volatile organic compounds are photochemically produced in the ocean surface microlayer, but estimates are missing. Here the authors combine experiments and observations to quantify photochemical emissions of volatile organic compounds and show that they are comparable to biological production.

## Introduction

Life on Earth is a constant and versatile source of surface-active compounds (i.e., surfactants) in the ambient environment. Biogenic surfactants are, therefore, omnipresent on surfaces exposed to the open atmosphere, ranging from lakes and rivers to cloud droplets, and aerosol particles^1^. Nonetheless, the most prominent example of a surfactant-enriched surface is probably the ocean, covering more than 70% of Earth. Studies have shown that this air/water interface is almost ubiquitously covered by a thin film of amphiphilic compounds, which are enriched there with respect to the bulk water^2–7^. Such sea surface microlayers (SMLs) were found to have significant effects on marine biogeochemical as well as climate related mechanisms by directly affecting processes such as exchange of trace gases (e.g., CO_2_), heat, and aerosol particles^3^. In addition, recent field studies^8,9^ confirmed for the first time previous laboratory observations^10–15^ suggesting that irradiation of this air/water interface by sunlight produces organic vapors, known to enhance particle formation in the atmosphere. These emissions were attributed to purely photochemical reactions occurring in the SML. However, current model calculations neglect this abiotic source of reactive compounds and account only for organic vapors that are produced directly by biological processes^16–20^.

Here, we combine for the first time results on the formation and presence of SMLs with observations of photochemical production of organic vapors from irradiation of surfactant-enriched air/water interfaces^10–15^, to identify locations and time periods in which such photochemistry is of major importance for marine VOC levels. Moreover, we explore the global extent of such yet underestimated VOC emissions as well as their implications for organic aerosol (OA) mass in marine environments. Our estimations are based on a collection of photochemical VOC production parameters, surface UV irradiation, biological activity, and surface wind speeds. Using these variables, we estimate SML coverage of the oceans and VOC formation potentials from interfacial photochemistry on a 1° × 1° global grid. We infer potential OA mass contributions from photochemically produced VOCs using aerosol partitioning theory and a one-dimensional volatility basis set approach^21–23^. As we demonstrate, accounting for abiotic photochemical VOC production closes current gaps in our understanding of atmospheric chemistry in remote regions and underlies the importance of the interplay between biology and chemistry for climatic processes. We infer global emissions of 32.5–129 Tg C yr^–1^ (23.2–91.9 TgC yr^–1^) of organic vapors from the oceans into the marine atmosphere. Moreover, our results suggest a potential contribution to organic aerosol mass of more than 60% over the remote ocean. In addition, to support planning and design of future field studies with a focus on VOC emissions from interfacial photochemistry, we provide global distributions of VOC emission potentials as well as a small calculation tool based on Matlab, to estimate photochemical VOC emissions depending on location and season.

## Results

### Global sea surface microlayer coverage

Since VOC formation from interfacial photochemistry will only occur in the presence of surfactants, our starting point is the global SML coverage of the oceans. The exact spatial and temporal dynamics of these surface films are largely dependent on local biological and meteorological conditions. In particular, high wind speeds at the ocean’s surface were shown to disturb the formation of such SMLs by breaking waves. The upper limit for the formation of SMLs was previously^2,24^ estimated to wind speeds of 8–10 m s^–1^, however, recent field observations^25^ suggest SML coverage even up to wind speeds of 13 m s^–1^.

**Fig. 1:**
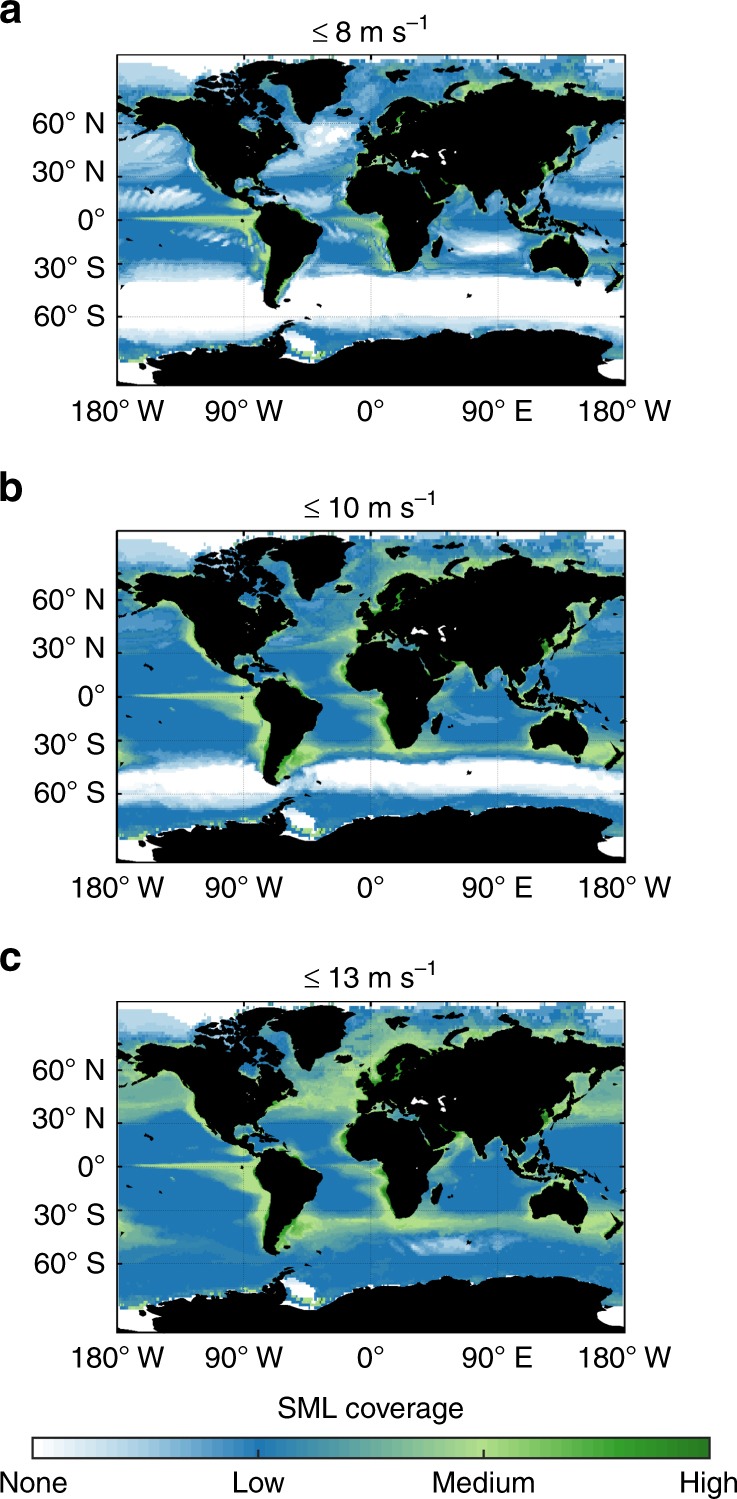
Average annual sea surface microlayer coverage of the surface of the oceans. **a**–**c** Demonstrate the effect of different wind speed limits on the predicted global surface coverage. In particular, the North Atlantic and North Pacific as well as the Southern Ocean react rather sensitive to the chosen threshold values

Following the approach of Wurl et al.^2^, we use biological net productivity and mean surface wind speeds to derive global SML distributions. As shown in Fig. 1, the largest effects for different wind speed thresholds are especially observed for the Southern Ocean, North Pacific, and North Atlantic. Our results indicate that these regions are largely free of SMLs for a wind speed limit of 8 m s^–1^ (Fig. 1a). In contrast, for a wind speed limit of 13 m s^–1^ almost the entire surface of the oceans can be regarded as covered at least partially by SMLs (Fig. 1c). A transition state between these two extremes, previously^2^ used to estimate the global presence of SMLs is shown in Fig. 1b.

### Photochemical potential of the surface of the oceans

Assuming a linear relationship between photochemical VOC production and solar radiation as well as a logarithmic decay with decreasing surfactant concentrations^10,11^, we can calculate a daily photochemical VOC emission potential *µ*_photo_ for the air/water interface of each grid cell:

1 $$\mu _{{\mathrm{photo}}} = t_{{\mathrm{sun}}} \cdot {\mathrm{UV}}_{{\mathrm{a}},{\mathrm{b}}} \cdot F_{{\mathrm{surfactant}}} \cdot k_{\mathrm{g}}$$

with the length of the day *t*_sun_ (in s), solar radiation UV_a,b_ at the Earth’s surface (280–400 nm, in mW cm^–2^ day^–1^), and a correction factor *F*_surfactant_ to account for SML presence and surfactant concentration. In addition, we add a factor *k*_g_ to account for the impact of wind on the gas transfer velocities across the air–sea interface, which are largely determined by sea-surface roughness^7^. We keep *µ*_photo_ in units of mW s cm^–2^ day^–1^ to allow for convenient conversion to daily VOC fluxes, as explained later. Here, we use averaged surfactant concentrations from field observations^2^ and normalize *µ*_photo_ to the highest surfactant concentrations, commonly observed in eutrophic regions. *F*_surfactant_ can therefore be expressed as

2 $$F_{{\mathrm{surfactant}}} = \frac{{{\mathrm{ln}}(c_{{\mathrm{surfactant}}})}}{{{\mathrm{ln}}(c_{{\mathrm{max}},{\mathrm{surfactant}}})}}$$

with the surfactant concentration *c*_surfactant_ (in concentration equivalents of Triton X), giving values between 0 and 1. For the gas transfer coefficient *k*_g_, we apply the wind speed dependent parameterization of McGillis and co-workers^26^ and normalize it to laboratory conditions of Ciuraru et al.^10^ and Brüggemann et al.^14^:

3 $$k_{{g}} = \frac{{8.2 + 0.014\cdot {U_{10}}^3}}{{8.2 + 0.014\cdot {U_{{\mathrm{lab}}}}^3}}$$

with the wind speed *U*_10_ at 10 m height, and the sample flow speed under laboratory conditions *U*_lab_ = 5.31 × 10^–2^ m s^–1^ (i.e., a flow rate of 200 mL min^–1^ scaled to a height of 10 m by assuming a log wind profile). Moreover, we perform all calculations at wind speed limits of 8, 10, and 13 m s^–1^ to account for the range of global SML abundances.

**Fig. 2:**
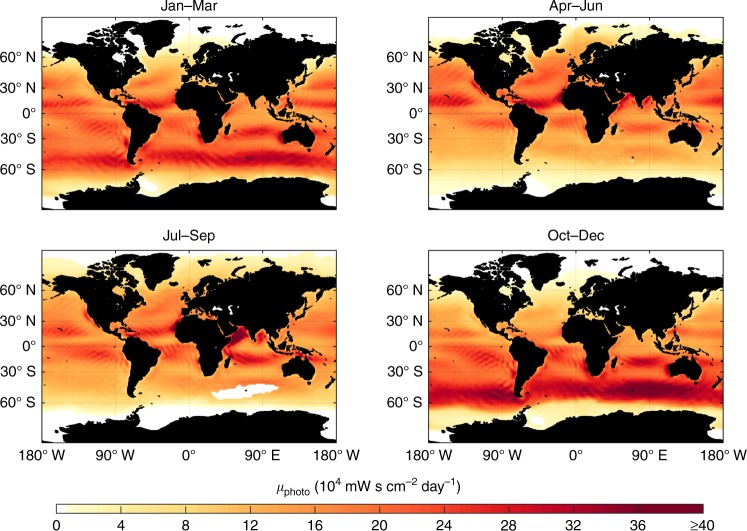
Seasonal VOC emission potential from interfacial photochemistry of biogenic surfactants. The depicted estimations assume a sea surface microlayer wind speed limit of 13 m s^–1^ (for wind speed limits of 8 and 10 m s^–1^, see Supplementary Figs. 1 and 2). Especially in remote marine regions with low microbiological activity, VOC formation from interfacial photochemistry is expected to play a significant role

As shown in Fig. 2, *µ*_photo_ and, thus, photochemical VOC formation is expected to occur especially in coastal waters in the tropics throughout the entire year. However, in these regions elevated direct biological VOC emissions are also anticipated due to high microbiological activity in surface waters. Thus, total annual photochemical VOC emissions are of minor importance in such locations, despite constantly high values for *µ*_photo_. Nonetheless, on shorter time scales interfacial photochemistry might play a pivotal role here, e.g., during the decay of microalgae blooms. In contrast, for remote regions of the oceans with high solar radiation but lower biological activity, interfacial photochemistry can serve as a major source of VOCs over longer periods. The extent of this abiotic source for organic vapors typically varies with the season. For example, the southern Indian Ocean exhibits an elevated *µ*_photo_ for the seasons Jan–Mar and Oct–Dec, whereas the remote North Pacific shows similar photochemical potentials for the seasons Apr–Jun and Jul–Sep. We note that the extent and variability of *µ*_photo_ eventually depends on the SML formation dynamics, and thus also on the wind speed limit for SML formation. For example, the high values obtained for the Southern Ocean for the season of Oct–Dec rapidly decline to zero, if we set the wind speed limit for SML formation to 10 m s^–1^ (Supplementary Fig. 2). To predict *µ*_photo_ with higher time and space resolution, it is therefore essential to resolve the factors affecting SML coverage in more detail in future laboratory and field studies.

**Fig. 3:**
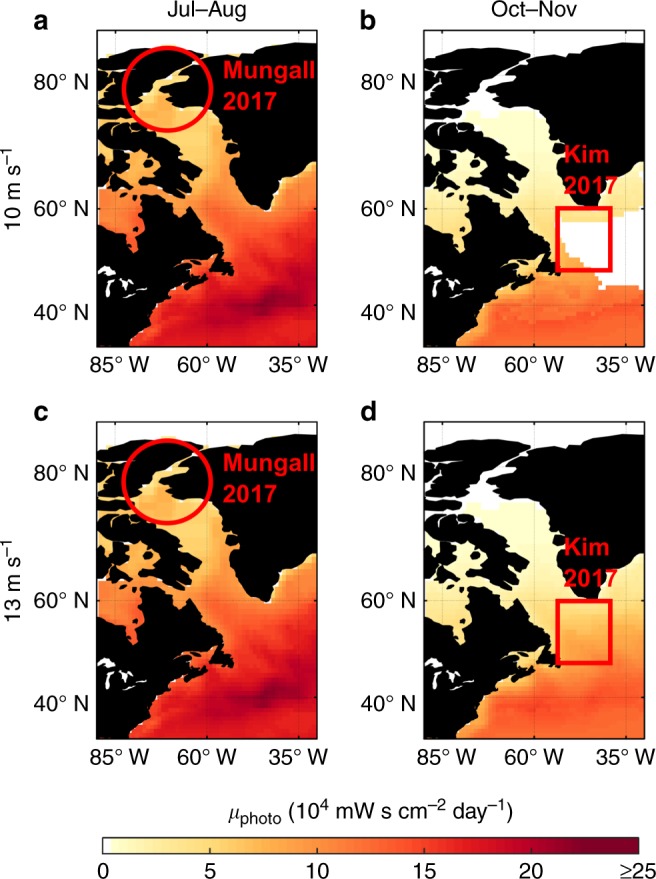
VOC formation potential *µ*_photo_. For wind speed limits of 10 m s^–1^ (**a**, **b**) and 13 m s^–1^ (**c**, **d**) for the locations and periods of the studies by Mungall et al.^8^ and Kim et al.^27^, i.e., for Jul–Aug and Oct–Nov in the western North Atlantic and Arctic

Up to now, field observations of photochemical VOC production from SMLs are scarce, which is certainly also due to the challenging differentiation between biologically and photochemically produced VOCs. However, Mungall et al. recently reported for the first time on interfacial photochemical production of oxygenated VOCs (OVOCs; e.g., formic acid, isocyanic acid, C_5_-oxo acids, etc.) in the Arctic during July–August^8^. In general, the observed OVOCs exhibited a higher degree of oxidation compared to laboratory studies. However, this difference might simply be due to some initial oxidation reactions of the originally emitted VOCs in the atmosphere, as sampling occurred ~15 m above the sea surface. Moreover, the applied acetate chemical ionization mass spectrometer is in particular sensitive towards acidic compounds, such as the detected organic acids. In addition to such OVOC emissions, Burkart et al. found enhanced new particle formation in the Arctic summer at low wind speeds and sunny conditions^9^. However, since new particle formation is a complex process, we note that besides interfacial photochemistry several other factors might have affected these observations significantly. Nevertheless, these field observations are in good agreement with our calculations, which show a maximum for *µ*_photo_ for the measurement location and time (indicated by the red circle in Fig. 3a and c). In contrast, Kim et al. found little to no evidence for isoprene formation from interfacial photochemistry for October–November in the same region^27^. This observation is, nonetheless, again in good agreement with our predictions, giving a minimal *µ*_photo_ for this location and season (indicated by the red square in Fig. 3b and d). As a conservative estimate, i.e., an SML wind speed limit of 10 m s^–1^ (Fig. 3, panels a and b), we estimate an average *µ*_photo_ of 6.88 × 10^4^ mW s cm^–2^ day^–1^ and 1.30 × 10^4^ mW s cm^–2^ day^–1^ for the study periods and regions of Mungall et al.^8^ and Kim et al.^27^, respectively. As can be seen from the figure, this large difference by a factor > 5 in *µ*_photo_ is mostly due to areas that are free of an SML under such conditions. The difference in *µ*_photo_ is, thus, decreasing when we allow the wind speed limit to increment to 13 m s^–1^ (Fig. 3, panels c and d). For these parameters the average *µ*_photo_ differs merely by a factor of about 1.2 for the two study periods and regions (i.e., 6.88 × 10^4^ and 5.67 × 10^4^ for Mungall et al.^8^ and Kim et al.^27^, respectively). However, it is important to note that Kim et al.^27^ observed high wind speeds of up to 25 m s^–1^ during their study period, with an average wind speed of 11 m s^–1^. Formation and presence of prevalent SMLs, and hence, photochemical VOC formation at the air/sea interface, are unlikely under such conditions. Thus, our calculations for an ocean surface that is largely free of SMLs (i.e., Fig. 3b) is probably better reflecting the actual study period.

Because of the demonstrated agreement between our predictions and these first field studies on interfacial photochemistry, we suggest utilizing *µ*_photo_ for future field campaigns to identify regions and time periods in which enhanced photochemical VOC production at the air/water interface can be expected. Moreover, to foster and support such studies in the ambient environment, we provide global distributions of *µ*_photo_ on a monthly basis for different SML wind speed limits (i.e., 8, 10, and 13 m s^–1^) in the Supplementary Information (Supplementary Figs 1 and 2) and added the source code to an online repository (see “Code availability” section).

### Marine isoprene emissions from interfacial photochemistry

**Fig. 4:**
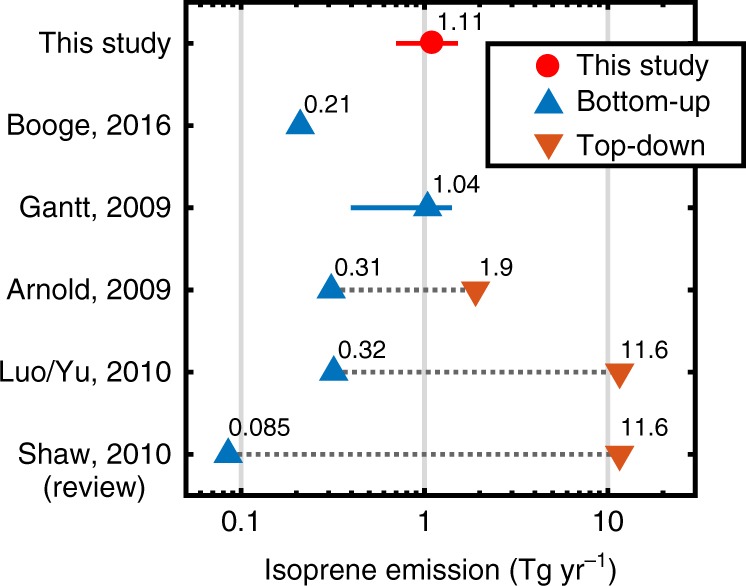
Comparison of current global isoprene emission estimates and the results of our study (0.70–1.52 Tg yr^−1^). The bottom-up estimates are based on direct biological emissions only, whereas top-down refers to expected total isoprene emissions from global model calculations

A further benefit of *µ*_photo_ is that multiplication with VOC production values from laboratory experiments (in molecules mW^–1^ s^–1^) directly yields estimates on VOC emissions (in molecules cm^–2^ day^–1^). Thus, allowing us to approximate photochemical VOC emission fluxes for a certain region and season, even for single compounds. As a simple example, we estimate global annual isoprene emissions of 1.11 (0.70–1.52) Tg yr^–1^ from interfacial photochemistry (Fig. 4), using SML wind speed limits of 8–13 m s^–1^ and photochemical production values from recent laboratory observations for authentic SML samples^10,11,14^. Emissions of other VOCs can easily be calculated in the same way. Comparison of this global flux to previously reported estimates on isoprene emissions demonstrates that interfacial photochemistry can compete, if not even surpass, direct biological emissions. Estimates from bottom-up studies, i.e., scaling up biological VOC emissions for different microorganism species, yield isoprene emissions in a similar range of 0.085–1.24 Tg yr^–1^. However, it was also shown that top-down approaches, i.e., calculating isoprene fluxes by atmospheric chemistry models, suggest much larger isoprene emissions of 1.9–11.6 Tg yr^–1^ globally^16,17,20,28,29^. Thus, accounting for interfacial photochemistry might aid in closing, or at least minimizing, this discrepancy between bottom-up and top-down approaches in global and regional models. In the same way, we infer total emissions of organic vapors from abiotic interfacial photochemistry in the range of 32.5–129 Tg C yr^–1^ (23.2–91.9 TgC yr^–1^), hence, contributing significantly to marine VOC emissions.

We note that the approach presented here can solely give an estimate on average monthly VOC fluxes from interfacial photochemistry. Thus, more sophisticated models with higher time and space resolution will be necessary, to predict photochemical VOC fluxes on a daily or even hourly basis. Nonetheless, as shown in Table 1, a direct comparison of observed and calculated isoprene fluxes demonstrates already a good agreement for single field studies, typically predicting somewhat lower fluxes than the reported ones. Remarkably, for almost all studies even the maximum predicted values do not exceed the range of observed isoprene emission fluxes. This general underprediction of isoprene emission fluxes compared to field studies is consistent with the assumption that interfacial photochemistry represents an additional VOC source besides direct biological emissions, significantly contributing to total marine VOC fluxes.

**Table 1:** Comparison of observed and calculated isoprene fluxes for single field studies

Field study	Comment	Location	Latitude^a^	Longitude	Period	Observed flux^b^	Calculated flux
						10^12^ molecules cm^−2^ day^−1^	
Hackenberg et al.^41^							
	AMT22	Atlantic	60° N–50° S	30° W	Oct–Nov	5.27 ± 3.97 (0.11–19.9)	4.18–11.1
	AMT23					3.80 ± 4.23 (0.0043–29.4)	
	ACCACIA1	North Atlantic/Arctic	70–77° N	15° W–15° E	Mar	0.59 ± 0.78 (0.035–4.32)	0.00–2.04
	ACCACIA2				Jul–Aug	2.59 ± 2.68 (0.026–12.1)	3.40–5.67
Kim et al.^27^		North Atlantic	55–60° N	44–54°W	Oct–Nov	4.32 ± 16.4	0.00–3.48
Tran et al.^c,^^42^							
	Wan	North Atlantic (Atl. water)	60–80° N	15° W–15° E	Jun–Jul	8.91 ± 23.4	4.56–7.60
	L&M					5.12 ± 12.3	
	Wan	Arctic (polar water)				2.53 ± 2.17	
	L&M					1.45 ± 1.20	
Meskhidze and Nenes^d,^^43^							
	SOFeX	Southern Ocean	40–60° S	20–55° W	Annual	5.18 (0.86–19.01)	0.10–10.4
	Palmer/Shaw					15.55 (1.73–74.30)	
Broadgate et al.^44^		North Sea	57° N	3° W	Annual	1.47 (0.09–4.06)	3.18–5.89
Milne et al.^45^		Florida Gulf	24° N	81° W	Sept	2.94 (0.57–6.05)	5.72–9.55
Baker et al.^46^		Eastern North Atlantic	53° N	13° W	May	2.94 (1.04–7.69)	0.00–12.1
Matsunaga et al.^47^		North Pacific	35° N	147° E	May	1.90–18.14	0.00–13.7
Sinha et al.^48^	Mesocosm experiment	North Sea	60° N	5° E	May–Jun	58.75 ± 11.08	4.82–8.05

^a^ Latitude and longitude area used for flux calculation

^b^flux mean ± standard deviation (range)

^c^Wan  =  flux parametrization from Wanninkhof^49^, L&M  =  flux parametrization from Liss and Merlivat^50^

^d^remotely sensed data, see original reference for method description

In particular, photochemical production and emission of larger (unsaturated) VOCs, such as isoprene, seems important, since few other abiotic formation pathways are known for such compounds in the marine environment. In contrast, the contribution to fluxes of smaller VOCs, such as acetone, or acetaldehyde, is probably less significant, since a large variety of strong sources exists in both the atmosphere and the ocean. As an example, Yang et al.^30^ observed emission fluxes of acetone and acetaldehyde of 7.11 × 10^14^ and 3.9 × 10^14^ molecules cm^–2^ day^–1^, respectively, during a cruise in the tropical Atlantic (3–39° N). However, our calculations merely predict emission fluxes in the range of 0.58–1.1 × 10^14^ and 0.20–0.60 × 10^14^ molecules cm^–2^ day^–1^ from interfacial photochemistry for acetone and acetaldehyde, respectively.

### Implications for marine organic aerosol mass

**Fig. 5:**
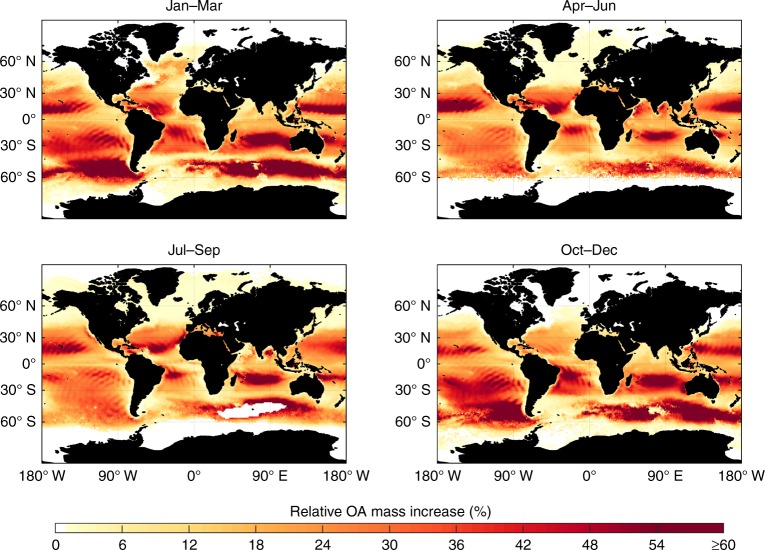
Seasonal estimates on the organic aerosol mass contribution in the marine boundary layer from oxidation of photochemically produced VOCs for an SML wind speed limit of 13 m s^–1^. For wind speed limits of 8 and 10 m s^–1^, see Supplementary Figs 5 and 6. Here, only OH oxidation of unsaturated volatile organic compounds was taken into account, assuming similar secondary organic aerosol yields as given by Tsimpidi et al.^23^

To assess possible implications of this yet unaccounted VOC source on OA mass loadings in the marine boundary layer, we calculate the formation of secondary OA (SOA) from unsaturated VOC oxidation products. To approximate background aerosol concentrations, primary OA emissions are estimated based on biological activity and surface wind speeds^17,18,31^ (Supplementary Fig. 3). As depicted in Fig. 5, our results indicate that especially in tropical regions with low POA concentrations additional SOA from oxidation of photochemically produced VOCs is important, contributing up to 60% of additional OA mass (e.g., in the Indian Ocean during Oct–Dec). Moreover, our calculations show a reasonable agreement with field studies in such remote regions (Supplementary Table 1). As an example, we obtain similar trends for OA mass concentrations as reported from long term observations on Amsterdam Island (37.8° S, 77.6° E; Supplementary Fig. 4)^32^. As already seen for the calculation of VOC fluxes, we typically observe a certain underprediction of total OA mass concentrations. This is, however, supporting again the hypothesis that SOA from photochemically produced VOCs is adding up on biologically derived SOA mass, which we omit in our calculations. In total, we estimate the additional annual SOA mass due to interfacial photochemistry to be in the range of 0.48–0.60 Tg yr^–1^. Oxidation products of photochemically produced isoprene are expected to contribute 33–73 Gg yr^–1^ to this total SOA mass, corresponding to about 0.2–0.5% of marine OA mass, which is in agreement with recent field observations^33,34^. Nonetheless, our estimations probably represent a lower limit, since we do not account for additional oxidation pathways of the formed VOCs, e.g., via ozone chemistry. In addition, we assume instantaneous mixing of the emitted VOCs into the entire volume of the MBL, probably leading to a rather diluted scenario compared to ambient conditions.

Despite remaining uncertainties, our study demonstrates that abiotic VOC production from interfacial photochemistry might serve as a major contributor to marine VOC levels and OA mass loadings. Isoprene emissions from photochemistry are expected to be in the same range as direct biological emissions and might even dominate depending on location and season. Moreover, SOA formation from oxidation of photochemically produced VOCs might explain field observations of new particle formation events which are occurring decoupled from biological activity. Therefore, we suggest that VOC and SOA production from interfacial photochemistry should be taken into account to accurately model and predict atmospheric chemistry and related processes, such as ozone cycling and cloud formation.

## Methods

### Software and data processing

All data processing was conducted using Matlab (version 8.6, R2015b, Mathworks Inc.), including the m_map tools package (v1.4h, https://www.eoas.ubc.ca/~rich/map.html) for the production of global maps. A general overview on all data sources and calculations is given in Supplementary Fig. 7. Moreover, the Matlab code as well as a stand-alone version of the Photochemistry At Liquid/Air interfaces containing Surfactants (PhotochemAtLAS) model, including instructions, is publicly available on Bitbucket, an online community repository.

### Production of organic vapors from interfacial photochemistry

For all calculations on photochemical VOC production presented here, we used the following three major assumptions. First, for the total surface area that is available for photochemistry, the air/water interface of the ocean is treated as a flat surface. We note that this simplification might eventually lead to an underestimation of VOC production, since it neglects increases in surface area, e.g., from waves, at the ocean’s surface. Nonetheless, since sea surface roughness is known to enhance gas diffusivity of the ocean’s air/water interface^7^, we estimate air–sea gas transfer velocities of VOCs as a function of wind speed, using the empirical parameterization of McGillis et al.^26^. In contrast to other common parameterization, this cubic *k*–*U*_10_ relationship is predicting non-zero emissions at zero wind speed, which is in agreement with laboratory studies on interfacial photochemistry and VOC production^10,11,14^. As shown in Supplementary Fig. 8, the wind speed-dependent gas transfer velocities can increase the calculated VOC fluxes by up to a factor of ~4.7 (i.e., in the Southern Ocean) compared to assuming constant gas transfer velocities across the air/sea interface. However, we note that there remains significant uncertainty of at least a factor of two in transfer velocities, as it is commonly the case for all trace gas emissions from the ocean into the atmosphere up to now^35,36^.

Second, photochemical VOC production correlates linearly with solar radiation, whereas a logarithmic decay with decreasing surfactant concentrations is observed^10,11^. This assumption is corroborated by measurements of Rossignol et al.^12^. who reported that absorbance at a specific wavelength is a linear function of the surfactant concentration. Moreover, Ciuraru et al.^10,11^. demonstrated that VOC production follows a logarithmic decay with decreasing surfactant concentrations. In addition, these assumptions are also consistent with the hypothesis that especially photosensitized reactions are driving VOC production from interfacial photochemistry^37^. Nonetheless, we note that these laboratory studies use single compounds as surfactants, whereas the ambient SML is a highly complex and dynamically changing composition of organic and inorganic compounds. Thus, we cannot exclude deviations for specific SML samples from these simplified assumptions.

Third, only radiation in the wavelength region of 280–400 nm initiates photochemical production of VOCs, since shorter wavelength might lead to photolysis, whereas the energy of longer wavelength is not sufficient to induce photochemical reactions. Furthermore, laboratory observations commonly used this wavelength range to infer photochemical production and fluxes of VOC^10–14^.

Given these constraints, we calculated a daily photochemical VOC emission potential *µ*_photo_ (in mW s cm^–2^ day^–1^) for the air/water interface of each 1-by-1 degree grid cell (Eq. (1)) with the length of the day *t*_sun_ (in s), solar radiation UV_a,b_ at the Earth’s surface (in mW cm^–2^ day^–1^), and a unitless correction factor *F*_surfactant_ to account for SML coverage and surfactant concentration (Eq. (2)). Moreover, we accounted for changes in gas transfer velocities across the air/sea interface by applying the wind speed-dependent factor *k*_g_, which we normalize to laboratory conditions (Eq. (3)). As explained further below, we keep *µ*_photo_ in units of mW s cm^–2^ day^–1^ to allow for convenient conversion to daily VOC fluxes.

As depicted in Supplementary Fig. 7, for *t*_sun_ and UV_a,b_ we used climatological data on solar irradiation at the surface of the Earth in the UV-a (315–400 nm) and UV-b (280–315 nm) range from the NCAR TUV model (https://www2.acom.ucar.edu/modeling/tropospheric-ultraviolet-and-visible-tuv-radiation-model), giving monthly averaged UV irradiance values (i.e., considering ozone, cloudiness, and *t*_sun_) for the period of 1979–2000. The data was refined from 1° × 1.25° resolution to 1° × 1° resolution by linear interpolation for all further calculations.

Global mapping of the ocean’s surfactant concentrations (*c*_surfactant_) and SML coverage was conducted according to the method described by Wurl et al.^2^. First, oligo-, meso- and eutrophic regions of the ocean were identified from global maps of net primary production (NPP), based on the Vertically Generalized Production Model^38^. Monthly data on NPP were obtained from the ocean productivity webpage of the Oregon State University (http://www.science.oregonstate.edu/ocean.productivity), ranging from July 2002 to November 2016 (Supplementary Fig. 9). After averaging the data for each month and lowering the resolution from 0.17° × 0.17° to 1° × 1° by linear interpolation, trophic states were defined as follows: oligotrophic waters with NPP < 0.4 g C m^–2^ day^–1^, mesotrophic waters with NPP between 0.4 and 1.2 g C m^–2^ day^–1^, and eutrophic waters with NPP > 1.2 g C m^–2^ day^–1^. Then, average surfactant concentrations were assigned to trophic states, based on mean values of Triton X equivalents (µg Teq L^–1^) from field observations^2^. The assigned surfactant concentrations are as follows: for oligotrophic waters *c*_surfactant_ = 320 µg Teq L^–1^ (i.e., low SML coverage), for mesotrophic waters *c*_surfactant_ = 502 µg Teq L^–1^ (i.e., medium SML coverage), and for eutrophic waters *c*_surfactant_ = 663 µg Teq L^–1^ (i.e., high SML coverage).

Furthermore, SML coverage was estimated for upper wind speed limits of 8, 10, and 13 m s^–1^. These values were reported earlier as maximum surface wind speed for SML formation^2,24,25^. Data on monthly mean surface wind speeds (January 1946–June 2016) were obtained from NCEP/NCAR reanalysis project (http://www.esrl.noaa.gov) and averaged for each month (Supplementary Fig. 10). Then, the monthly data were refined from 2.5° × 2.5° resolution to 1° × 1° resolution by linear interpolation. All regions exceeding the selected wind speed limit were considered as free of SMLs and, thus, considered as photochemically not active.

For our estimations, we normalized *µ*_photo_ to the highest surfactant concentrations *c*_max,surfactant_, as observed in eutrophic regions (i.e., 663 µg Teq L^–1^). Furthermore, we assumed photochemical VOC production to exhibit a logarithmic decay with decreasing surfactant concentrations^10,11^. *F*_surfactant_ was therefore expressed as shown in Eq. (2).

The effect of this correction factor on photochemical VOC production is illustrated in Supplementary Fig. 11, demonstrating reduced VOC production at lower surfactant concentrations^10,11^. For the lowest surfactant concentration of 320 µg Teq L^–1^, commonly observed in oligotrophic regions^2^, *F*_surfactant_ will reduce the estimated photochemical VOC emissions by ca. 11%. For mesotrophic conditions, i.e., *c*_surfactant_ = 502 µg Teq L^–1^, a reduction of about 4% is expected. We stress that up to now this correction is solely based on laboratory studies for single compounds, whereas the ambient SML is a complex mixture of organic and inorganic compounds. Therefore, we suggest focusing in future studies on more complex mixtures of surfactants, ambient SML samples, and also the relationship between surface pressure and VOC production.

To infer estimates on photochemical VOC production and emission of single compounds from interfacial photochemistry at the surface of the ocean, *µ*_photo_ is multiplied by data from VOC flux measurements in laboratory studies:

4 $${\rm{VOC}}_{{\rm{ambient}}} = {\rm{VOC}}_{{\rm{lab}}} \cdot \mu _{{\rm{photo}}}$$

with the estimated VOC emissions VOC_ambient_ (in molecules cm^–2^ day^–1^), measured photochemical VOC emissions from laboratory studies VOC_lab_ (in molecules mW^–1^ s^–1^), and the photochemical potential *µ*_photo_. Values for VOC_lab_ can readily be obtained from photochemical experiments under controlled conditions, using common VOC measurement techniques such as proton transfer reaction mass spectrometry (PTR-MS). In this study we used such data from previous work of George and co-workers^10,11,14^. For example, to estimate global emissions of isoprene from interfacial photochemistry, we used previously reported values for VOC_lab_ from marine SML and biofilm samples^10,11,14^, which are in the range of 3.71–6.19 × 10^7^ molecules mW^–1^ s^–1^.

### Estimating secondary organic aerosol formation

As depicted in Supplementary Fig. 7, the VOC module described above is coupled to an SOA module, which we utilized to retrieve estimates on potential SOA formation from photochemically produced and emitted VOCs. This module is based on equilibrium partitioning theory^21^ and a one-dimensional volatility basis set^22^, lumping VOC oxidation products in 10 volatility bins with saturation vapor pressures of log C*(300 K) = {−3, −2, −1, 0, 1, 2, 3, 4, 5, 6} µg m^–3^. Since so far no parameterization of SOA yields for oxidation of VOCs from interfacial photochemistry is available, here we accounted solely for oxidation of unsaturated VOCs by OH radicals, using SOA yields for alkenes previously given by Tsimpidi et al.^23^ (i.e., OLE2-case at low NO_X_ concentrations; *α* = {0, 0, 0, 0.023, 0.044, 0.129, 0.375, 0, 0, 0}). Moreover, we assumed pseudo-first order kinetics for the reaction of VOCs with OH radicals. We attempted to approximate the reaction rate constant *k*_OH_ for the mixture of unsaturated VOCs by averaging known *k*_OH_ constants of similar compounds^39^. An overview of the compounds and rate constants used for this approximation is given in Supplementary Table 2. Using these values we found a mean reaction rate constant of *k*_OH_ = 109.76 × 10^12^ cm^3^ molecule^–1^ s^–1^, which was then applied to estimate formation of VOC oxidation products. For all calculations, we used an OH concentration of *c*_OH_ = 1.5 × 10^6^ molecules cm^–3^. Unless stated otherwise, the aging time for SOA formation and growth was set to 12 h per day at a temperature of 15 °C.

In addition, we estimated primary organic aerosol (POA) mass fluxes and concentrations above the ocean, in order to model accurately the partitioning of VOC oxidation products. POA was, however, solely regarded as background aerosol, which was not taking part in partitioning, i.e., POA was defined as non-volatile. Here we applied established POA emission schemes, which infer POA fluxes from sea surface temperature, chlorophyll-a concentration, and surface wind speed^18,39^. Monthly mean data on sea surface temperature and chlorophyll-a concentrations were obtained from MODIS ocean products (https://modis.gsfc.nasa.gov/). POA emissions were instantaneously diluted into the marine boundary layer (MBL). Monthly mean MBL heights were obtained from the NOAA-CIRES 20th Century Reanalysis project (https://www.esrl.noaa.gov/psd/data/20thC_Rean/) (Supplementary Fig. 12).

As a simplified example, Supplementary Fig. 13 shows the result of a simulation of VOC formation and oxidation, as well as SOA particle formation for one grid cell. In this case, the following parameters were used: a POA mass concentration of 0.4 µg m^–3^ (i.e., representing typical remote marine air^40^), an ambient temperature of 20 °C, an MBL height of 600 m, and an emission rate of unsaturated VOCs of VOC_ambient_ = 4.1×10^9^ molecules cm^–2^ s^–1^ (i.e., representing tropical regions). Both VOC formation from interfacial photochemistry and oxidation of the gas-phase products start at time 0. The resulting mixing ratio of, yet unreacted, unsaturated VOCs is depicted in the left panel of the figure, reaching a steady state of ca. 4 × 10^8^ molecules cm^–3^ (i.e., 16 ppt_V_) after 8 h. This equilibrium mixing ratio might seem quite low, however, we note that here we assume instantaneous mixing of the emitted VOCs into the entire volume of the MBL. Therefore, ambient mixing ratios in direct proximity of the air/water interface (i.e., a few meters above the surface of the ocean) are likely to be much larger, whereas our calculations solely represent a lower limit.

Nonetheless, even for this dilute scenario we observe significant SOA formation from VOC oxidation products, as depicted in the right panel of Supplementary Fig. 13. Despite direct formation of VOC oxidation products, for the first 2 h almost no increase in OA mass concentration is observed. This delayed increase in particle mass is due to the enhanced partitioning of oxidation products to the gas phase until a critical gas-phase concentration is reached. After this initial period, low volatile oxidation products start to partition into the particle phase, resulting in an increase in OA particle mass concentration. This increase is then accelerating with ongoing formation of VOC oxidation products. In this simplified example, eventually a total increase of 0.088 µg m^–3^ is observed after a total aging time of 12 h, i.e., a relative increase of 22% in OA particle mass. Again, this value represents solely a lower limit of enhanced SOA formation, since we excluded ozone chemistry, which would generate additional low volatility oxidation products, probably enhancing OA particle mass concentrations.

### Code availability

The Matlab code of the Photochemistry At Liquid/Air interfaces containing Surfactants (PhotochemAtLAS) model, including instructions, is publicly available on the online community repository Bitbucket (https://bitbucket.org/bruggemann_m/photochematlas).

### Data availability

Further data sets generated during this study are available from the corresponding author upon reasonable request.

## Electronic supplementary material

Supplementary Information
