# Author Correction: Interfacial photochemistry at the ocean surface is a global source of organic vapors and aerosols

**DOI:** 10.1038/s41467-018-05687-3

**Published:** 2018-08-08

**Authors:** Martin Brüggemann, Nathalie Hayeck, Christian George

**Affiliations:** 1 Univ Lyon, Université Claude Bernard Lyon 1, CNRS, IRCELYON, F-69626 Villeurbanne, France; 2 Present Address: Leibniz Institute for Tropospheric Research (TROPOS), Permoserstr. 15, 04318 Leipzig, Germany

Correction to: *Nature Communications* 10.1038/s41467-018-04528-7; published online 23 April 2018

The authors became aware of a mistake in the data displayed in the original version of the paper. Specifically, for the calculation of the total emission estimates (i.e., from an average molecular weight and summed laboratory production values for all VOCs), the authors mistakenly added seasonal estimates to the annual estimates because both values are stored in the same variable of the code. Eventually, this additional sum resulted in a doubling of emission estimates.

As a result of this, the following changes have been made to the originally published version of this Article:

The fifth sentence of the abstract originally read “Our results indicate global emissions of 46.4–184 Tg C yr^–1^ of organic vapors from the oceans into the marine atmosphere and a potential contribution to organic aerosol mass of more than 60% over the remote ocean.” In the corrected version “46.4–184 Tg C yr^–1^” is replaced by “23.2–91.9 Tg C yr^-1^”

The seventh sentence of the second paragraph of the Introduction originally read “We infer global emissions of 65.0–257 Tg yr^–1^ (46.4–184 Tg C yr^–1^) of organic vapors from the oceans into the marine atmosphere.” In the corrected version, “65.0–257 Tg yr^–1^ (46.4–184 Tg C yr^–1^)” is replaced by “32.5–129 Tg C yr^−1^ (23.2–91.9 Tg C yr^-1^)”.

The last sentence of the first paragraph of the Results subheading “Marine isoprene emissions from interfacial photochemistry” originally read “In the same way, we infer total emissions of organic vapors from abiotic interfacial photochemistry in the range of 65.0–257 Tg yr^–1^ (46.4–184 Tg C yr^–1^), hence, contributing significantly to marine VOC emissions.” In the corrected version, “65.0–257 Tg yr^–1^ (46.4–184 Tg C yr^–1^)” is replaced by “32.5–129 Tg C yr^−1^ (23.2–91.9 Tg C yr^−1^)”.

This has been corrected in both the PDF and the HTML versions of the Article. While the new estimates are lower than previously reported this error does not affect the original discussion or conclusions of the Article. The authors apologize for the confusion caused by this mistake.

