# Supplementary material for: Interfacial photochemistry at the ocean surface is a global source of organic vapors and aerosols
Source: Nat Commun. 2018 May 29;9:2101. doi: 10.1038/s41467-018-04528-7 (PMC5974316; doi:10.1038/s41467-018-04528-7)
Supplement: Supplementary file 1 — Supplementary Information [file 41467_2018_4528_MOESM1_ESM.pdf]

Supplementary Information to

Interfacial photochemistry at the ocean surface is a global source of organic vapors and aerosols

Martin Brüggemann[1,2], Nathalie Hayeck[1], Christian George[1,*]

[1]Univ Lyon, Université Claude Bernard Lyon 1, CNRS, IRCELYON, F-69626, Villeurbanne, France

[2]now at: Leibniz Institute for Tropospheric Research (TROPOS), Permoserstr. 15, 04318 Leipzig, Germany

*correspondence to:

Dr. Christian George
CNRS – IRCELYON
2 avenue Albert Einstein
69626 Villerbanne Cedex
France

+33 (0) 4 72 44 81 90
christian.george@ircelyon.univ-lyon1.fr

List of Figures and Tables:

page

Supplementary Figure 1: Seasonal VOC formation potential from interfacial photochemistry of biogenic surfactants. The depicted estimations assume an SML wind speed limit of 8 m s^{-1}. 3

Supplementary Figure 2: Seasonal VOC formation potential from interfacial photochemistry of biogenic surfactants. The depicted estimations assume an SML wind speed limit of 10 m s^{-1}. 4

Supplementary Figure 3: Annual average of calculated marine POA mass concentrations, based on sea surface temperature, chlorophyll-a concentration, and surface wind speed[1,2]. Monthly mean data on sea surface temperature and chlorophyll-a concentrations were obtained from MODIS ocean products (https://modis.gsfc.nasa.gov/) for the period 07/2002–04/2017. POA emissions were instantaneously diluted into the marine boundary layer (MBL). 5

Supplementary Table 1: Observed and calculated clean marine organic aerosol concentrations (µg m^{-3})*. Calculations were conducted for laboratory values on unsaturated VOCs from Ciuraru *et al.*[3], using an SML wind speed limit of 13 m s^{-1}. In addition, the relative increase in OA mass from oxidation of photochemically formed VOCs is given. 6

Supplementary Figure 4: Comparison of observed[4] and calculated OA mass concentrations at Amsterdam Island (37.8 °S, 77.6 °E). The calculations suggest that especially during November and April POA and VOC oxidation products from abiotic photochemistry are the main contributors to OA levels. 8

Supplementary Figure 5: Seasonal estimates on the OA mass contribution in the marine boundary layer from oxidation of photochemically produced VOCs for an SML wind speed limit of 8 m s^{-1}. 9

Supplementary Figure 6: Seasonal estimates on the OA mass contribution in the marine boundary layer from oxidation of photochemically produced VOCs for an SML wind speed limit of 10 m s^{-1}. 10

Supplementary Figure 7: Overview on data resources and calculations conducted using the Photochemistry At Liquid/Air interfaces containing Surfactants (PhotochemAtLAS) model. 11

Supplementary Figure 8: a) Normalized gas transfer velocities k_g, using the empirical k–U_{10} parameterization of McGillis *et al.*[5] b) Annual distribution of normalized gas transfer velocities k_g. The strongest effect (up to a factor of 4.7) is observed in the Southern Ocean, where elevated wind speeds enhance air–sea gas transfer velocities (see also Supplementary Fig. 12). 12

Supplementary Figure 9: Annual average of net primary productivity, calculated from monthly means from Jul 2002–Nov 2016. Data were obtained from the Ocean Productivity webpage of the Oregon State University (http://www.science.oregonstate.edu/ocean.productivity). 13

Supplementary Figure 10: Annual mean wind speeds, calculated from monthly mean wind speeds at 10 meters above the surface of the ocean. Data were obtained from the NCEP/NCAR Reanalysis dataset (https://www.esrl.noaa.gov/). 14

Supplementary Figure 11: Correction factor as a function of surfactant concentration. According to Ciuraru et al. a logarithmic behavior of VOC emissions and surfactant concentration is expected[3,6]. 15

Supplementary Table 2: List of unsaturated VOCs produced by interfacial photochemistry (identified in laboratory experiments by online-APCI–MS[7]). Reaction rate constants with OH were taken from Atkinson and Arey[8]. For compounds with unknown kOH a chemically similar proxy compound was selected. 16

Supplementary Figure 12: Annual mean MBL heights. Data were obtained from the NOAA-CIRES 20th Century Reanalysis project (https://www.esrl.noaa.gov/psd/data/20thC_Rean/) for the period 1981–2010. 17

Supplementary Figure 13: SOA formation within one grid cell from oxidation of VOC produced from interfacial photochemistry. Photochemistry and OH oxidation are started in parallel at time 0. The left panel shows the mixing ratio of yet unreacted unsaturated VOCs from interfacial photochemistry. The right panel shows the increase in OA mass concentration and the total mass of oxidation products (i.e., gas and particle phase combined). 18

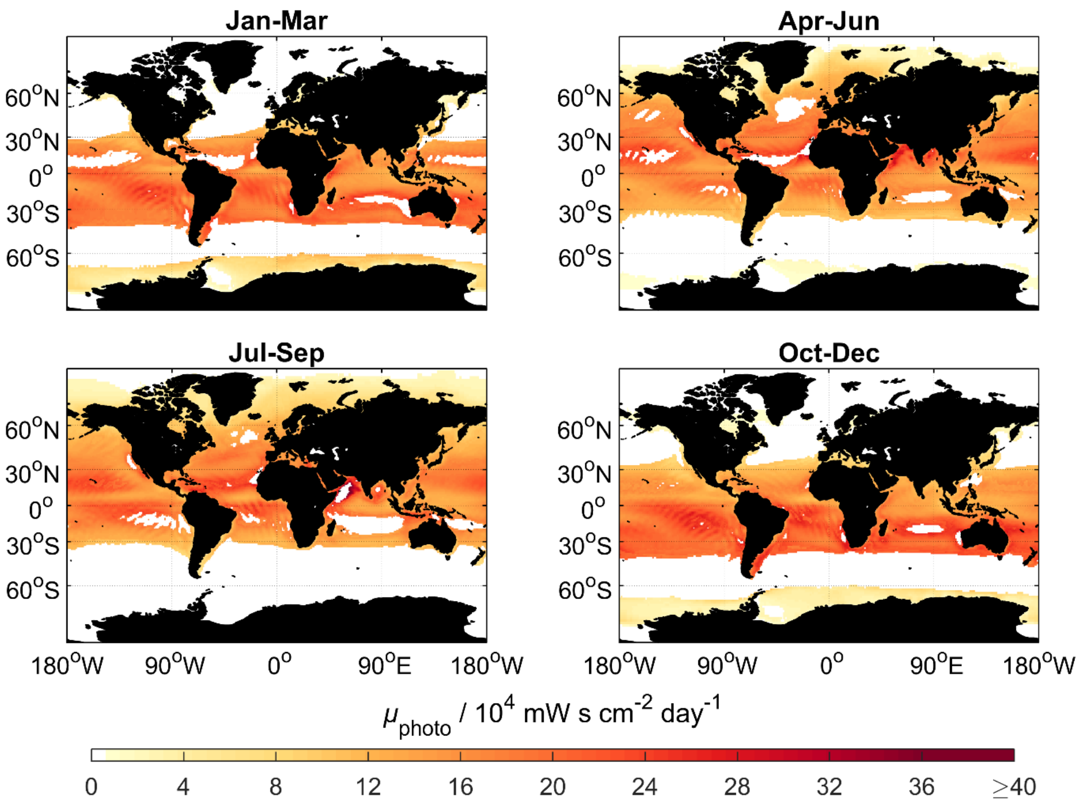

Supplementary Figure 1 | Seasonal VOC formation potential from interfacial photochemistry of biogenic surfactants. The depicted estimations assume an SML wind speed limit of 8 m s^{-1}.

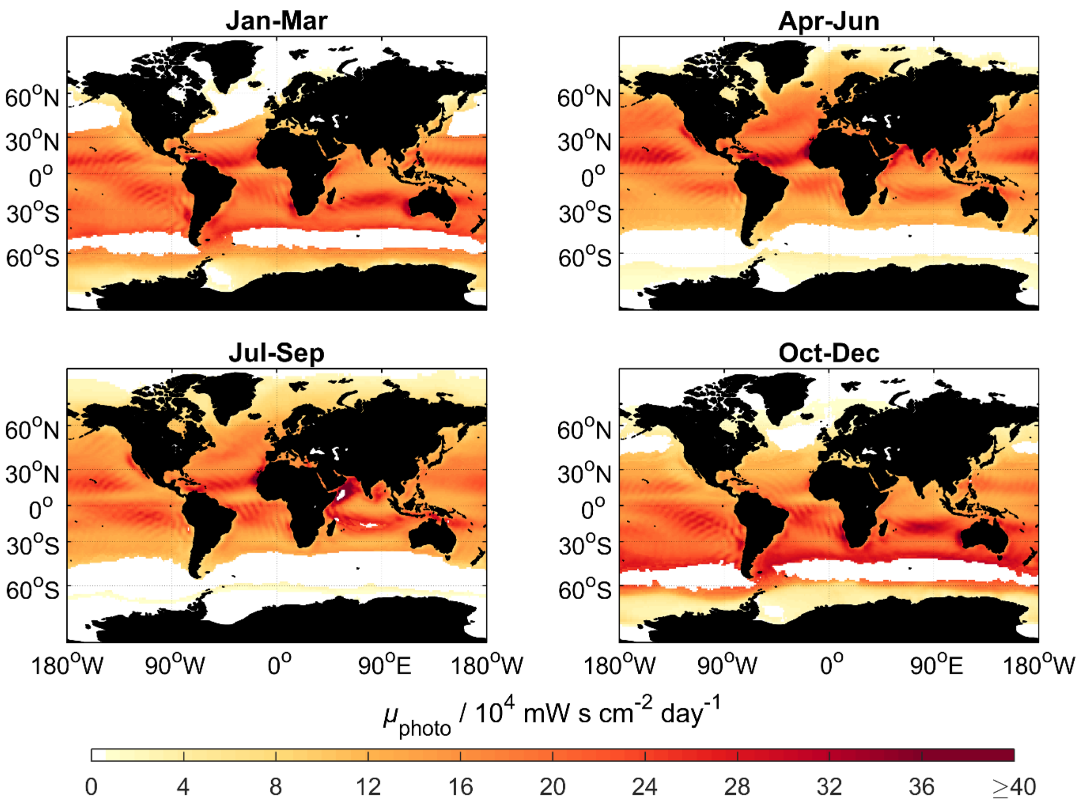

Supplementary Figure 2 | Seasonal VOC formation potential from interfacial photochemistry of biogenic surfactants. The depicted estimations assume an SML wind speed limit of 10 m s^{-1}.

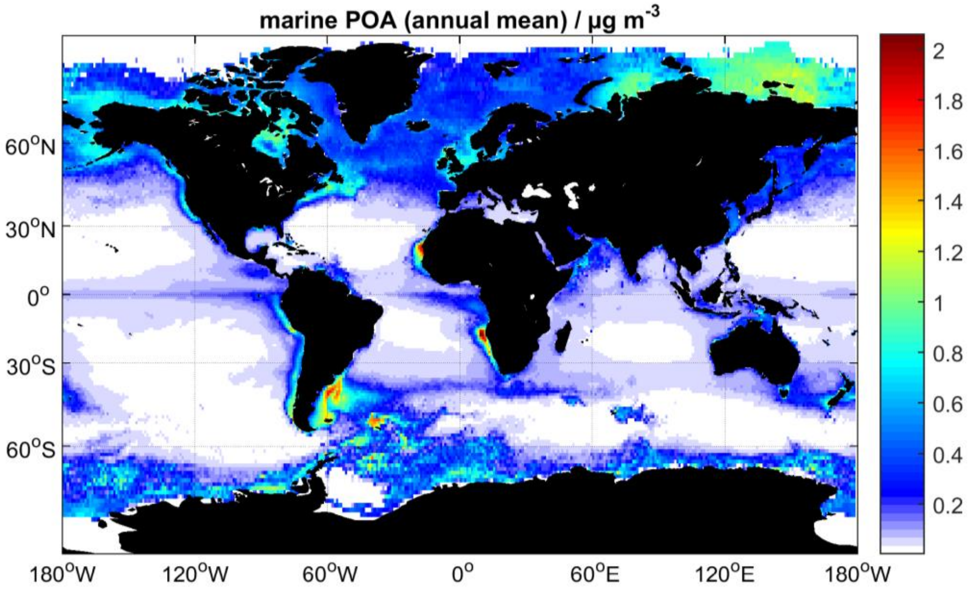

Supplementary Figure 3 | Annual average of calculated marine POA mass concentrations, based on sea surface temperature, chlorophyll-a concentration, and surface wind speed[1,2]. Monthly mean data on sea surface temperature and chlorophyll-a concentrations were obtained from MODIS ocean products (https://modis.gsfc.nasa.gov/) for the period 07/2002–04/2017. POA emissions were instantaneously diluted into the marine boundary layer (MBL).

Supplementary Table 1 | Observed and calculated clean marine organic aerosol concentrations (μg m^{-3})*. Calculations were conducted for laboratory values on unsaturated VOCs from Ciuraru *et al.*[3], using an SML wind speed limit of 13 m s^{-1}. In addition, the relative increase in OA mass from oxidation of photochemically formed VOCs is given.

reference	note	Location	Lat	Lon	Collection date	OA concentration / μg m^{-3}		relative increase / %
						observed	calculated	
Barger and Garrett (1976)	*(A), (B), (C)*	Eastern Tropical Pacific	5.49	-83.72	11-12 Feb 1974	0.82	0.13	6.8
			3.49	-87.56	12-13 Feb	0.24	0.10	8.0
			1.74	-89.42	13-14 Feb	0.57	0.12	8.6
			1.28	-89.94	14-15 Feb	0.59	0.15	7.5
			1.94	-92.49	15-16 Feb	0.57	0.16	6.9
			1.53	-93.98	16-17 Feb	0.16	0.16	7.0
			0.48	-92.47	17-18 Feb	0.08	0.23	5.9
			0.23	-89.92	18-19 Feb	0.5	0.19	6.6
			1.32	-87.35	19-20 Feb	0.16	0.18	6.5
			2.43	-84.84	20-21 Feb	0.24	0.16	6.3
			1.83	-82.87	21-22 Feb	0.41	0.13	7.7
			1.52	-81.75	22-23 Feb	0.51	0.13	7.5
Hoffman and Duce (1977)	*(A)*	Hawaii	21.37	-147.7	Jul-Oct 1975	0.54	0.026	31
Hoffman and Duce (1977)	*(D)*	American Samoa	-14.3	-170.57	Jun-Aug 1976	0.31	0.037	25
Andreae (1983)	*(C)*	Northeast Atlantic	40	-10	Oct 1980	0.42	0.56	1.2
Andreae (1983)	*(C)*	Western Tropical Atlantic	-20	-40	Nov 1980	0.43	0.071	17
Andreae et al. (1984)	*(B), (E), (F), (G)*	Tropical Pacific	0	-110	Jul 1982	0.35	0.20	7.5
			0	-115	Jul	0.24	0.19	7.6
			0	-122	Jul	0.21	0.16	8.4
			-2	-140	Jul	0.11	0.079	12
			0	-140	Jul	0.17	0.12	8.4
			10	-140	Aug	0.39	0.052	13
			18	-140	Aug	0.39	0.026	44
Cachier et al. (1986)	*(H)*	American Samoa	-14.3	-170.57	7 Jul 1981	0.18	0.037	25
					17 Jul 2017	0.15	0.037	25
					28 Jul 2017	0.15	0.037	25
					5 Aug 2017	0.1	0.038	30
					26 Jan 2017	0.13	0.044	23
					17 Feb 2017	0.2	0.045	19
Cachier et al. (1986)	*(H)*	Amsterdam Island	-37.8	77.6	10 Feb 1982	0.21	0.11	9.4
Putaud et al. (2000)	*(I), (J)*	Tenerife	28.3	-16.5	Jun-Jul 1997	0.29	0.050	48
Quinn et al. (2004)	*(K), (L), (M)*	Northeast Pacific	30	-160	1 Mar 2001	0.28	0.042	20
Zorn et al. (2008)	*(B), (O)*	Southwest Atlantic	-44	-20	1 Jan 2007	0.03	0.081	22
			-44	-60	31 Jan-2 Feb	0.32	1.2	24
			-60	-40	Mar	0.02	0.072	8.1
Sciare et al. (2009)	*(P), (Q)*	Amsterdam Island	-37.8	77.57	Jan 2003-2007	0.381	0.13	10.3
					Feb	0.253	0.11	9.4
					Mar	0.203	0.11	8.0
					Apr	0.172	0.17	6.4
					May	0.171	0.078	7.7
					Jun	0.136	0.050	10
					Jul	0.144	0.033	19
					Aug	0.15	0.032	27
					Sep	0.164	0.037	38
					Oct	0.13	0.084	19
					Nov	0.151	0.15	11

					Dec	0.272	0.17	8.8
Lee et al. (2010)	*(F), (J), (N)*	Eastern Tropical Atlantic	29	-15	1 May 2007	0.2	0.053	33
			23	-21	May	0.8	0.14	11
			21	-22	May	0.6	0.78	2.4
			19	-25	May	0.4	0.053	30
Russell et al. (2010)	*(F), (J)*	Northeast Atlantic	46.49	-44.67	1 Mar 2008	0.73	0.55	1.4
			70.95	-7.8	Apr	0.27	0.22	0.9
			70.24	-13.4	Apr	0.28	0.11	1.8
Miyazaki et al. (2010)	*(B), (R), (S)*	Northwest Pacific	41	155	Jul-Aug 2008	0.88	0.079	4.9
	(B), (R), (T)		45	167	Aug	0.55	0.28	1.3
Miyazaki et al. (2011)	*(B), (R)*	Northwest Pacific	30	150	1 Sep 2008	1.06	0.026	19
	(B), (R)		43	155	Aug	1.69	0.085	4.3
Fu et al. (2011)	*(B), (R), (U)*	North Pacific	31	175	1 Nov 1989	0.22	0.026	16
			30	-160	Nov	0.08	0.029	16
			32	-135	Nov	0.11	0.039	9.9
(27) Fu et al. (2011)	*(B), (R), (U)*	North Atlantic	30	-60	1 Dec 1989	0.32	0.031	13
			30	-35	Dec	0.25	0.034	12
Shank et al. (2012)	*(J), (O)*	Northeast Pacific	45	-130	1 Apr 2006	0.15	0.20	2.7
Shank et al. (2012)	*(J), (O)*	Eastern Tropical Pacific	-20	-85	Oct-Nov 2008	0.1	0.048	36
Shank et al. (2012)	*(J), (O)*	Eastern Tropical Pacific	10	-140	1 Sep 2009	0.02	0.049	12

* When applicable, concentrations are converted from µg C m^{-3} to µg m^{-3} using an organic matter/organic carbon ratio of 1.4.

(A) Atmospheric samples were collected over a period of 24 to 48 hours on precombusted glass-fiber filters and extracted with chloroform
(B) Ship-borne measurements with the average coordinates reported between the start and end of a sampling period
(C) We have included samples having BC concentrations < 0.05 µg m-3 and a BC/total carbon ratio < 0.1
(D) Atmospheric samples were collected over a period of 7 days on precombusted glass fiber filters and extracted with chloroform
(E) In the aerosol fraction ≤ 1.7 µm diameter
(F) Samples having BC concentrations < 0.05 µg m^{-3}
(G) Excess potassium < 5 ng m^{-3}
(H) Atmospheric samples were collected over a period of 2 days to 2 weeks on precombusted glass-fiber filters and extracted with different kinds of solvent
(J) In the aerosol fraction ≤ 1 µm diameter
(K) Atmospheric samples were collected over a period of 2.5 to 12 hours on quartz filter and analyzed with a Sunset Labs thermal/optical analyzer
(L) In the aerosol fraction ≤ 1.1 µm diameter
(M) The mass of particulate organic matter (POM) was determined by multiplying the measured OC concentration in µg m^{-3} by a factor of 2.1 in the marine region
(N) The real-time quantitative measurements of atmospheric samples were conducted using Aerodyne Aerosol Mass Spectrometer (AMS)
(O) The real-time quantitative measurements of atmospheric samples were conducted using Aerodyne High-Resolution-Time-of-Flight AMS (HR-ToF-AMS)
(P) Atmospheric samples were collected over a period of 8 to 10 days on prefired Whatman QMA quartz filters and analyzed with a thermo-optical transmission carbon analyzer system and total organic carbon analyzer
(Q) Non-methanesulfonate organic carbon (nms-OC)
(R) Atmospheric samples were collected on precombusted quartz filters and analyzed using a Sunset lab EC/OC analyzer
(S) Highly biologically influenced aerosols
(T) Less biologically influenced aerosols
(U) Samples having EC concentrations below detection limit (not defined) are used

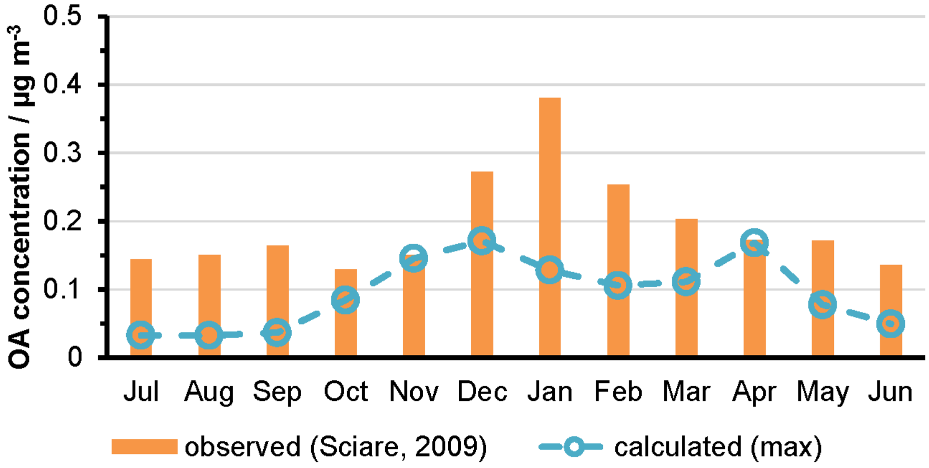

Supplementary Figure 4 | Comparison of observed[4] and calculated OA mass concentrations at Amsterdam Island (37.8 °S, 77.6 °E). The calculations suggest that especially during December and April POA and VOC oxidation products from abiotic photochemistry are the main contributors to OA levels.

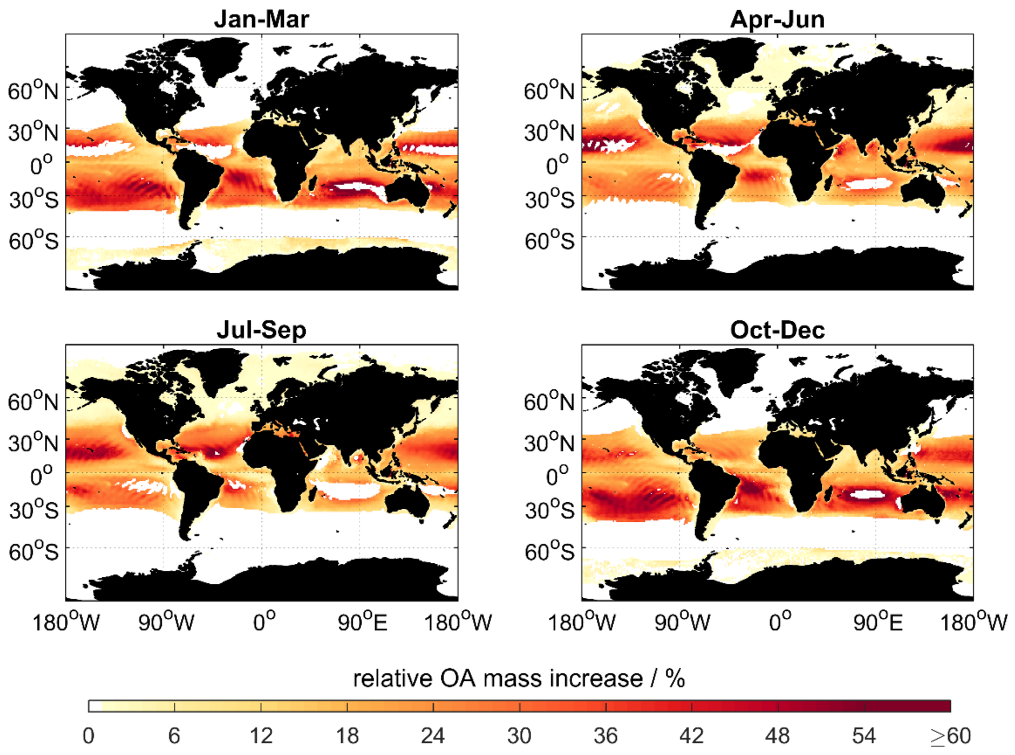

Supplementary Figure 5 | Seasonal estimates on the OA mass contribution in the marine boundary layer from oxidation of photochemically produced VOCs for an SML wind speed limit of 8 m s^{-1}.

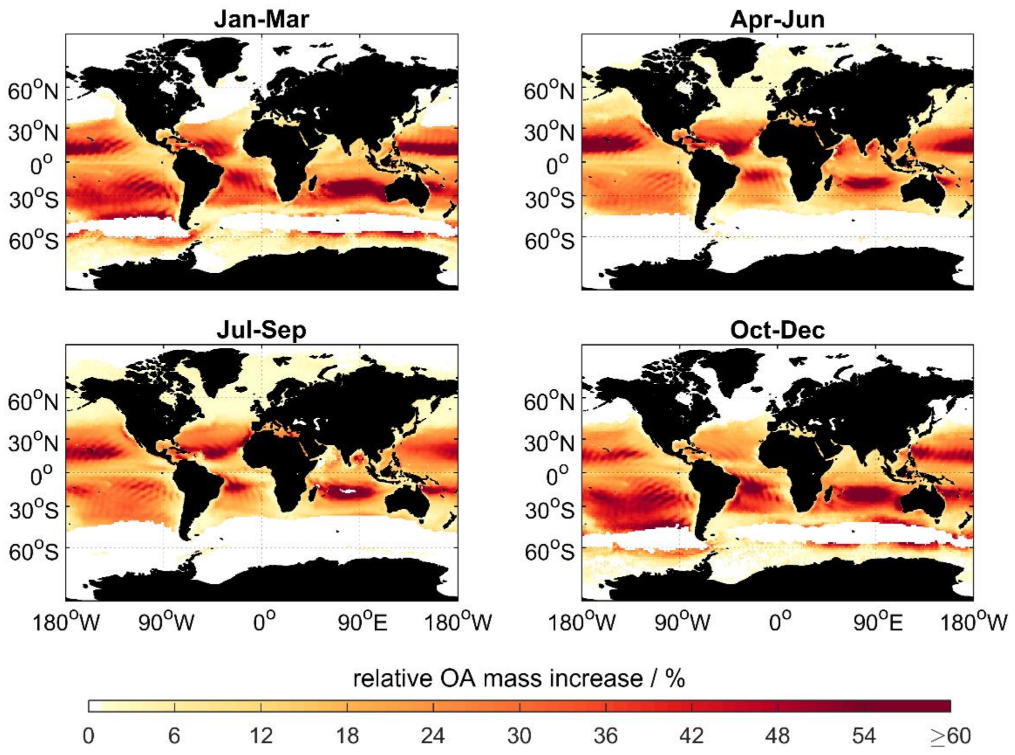

Supplementary Figure 6 | Seasonal estimates on the OA mass contribution in the marine boundary layer from oxidation of photochemically produced VOCs for an SML wind speed limit of 10 m s^{-1}.

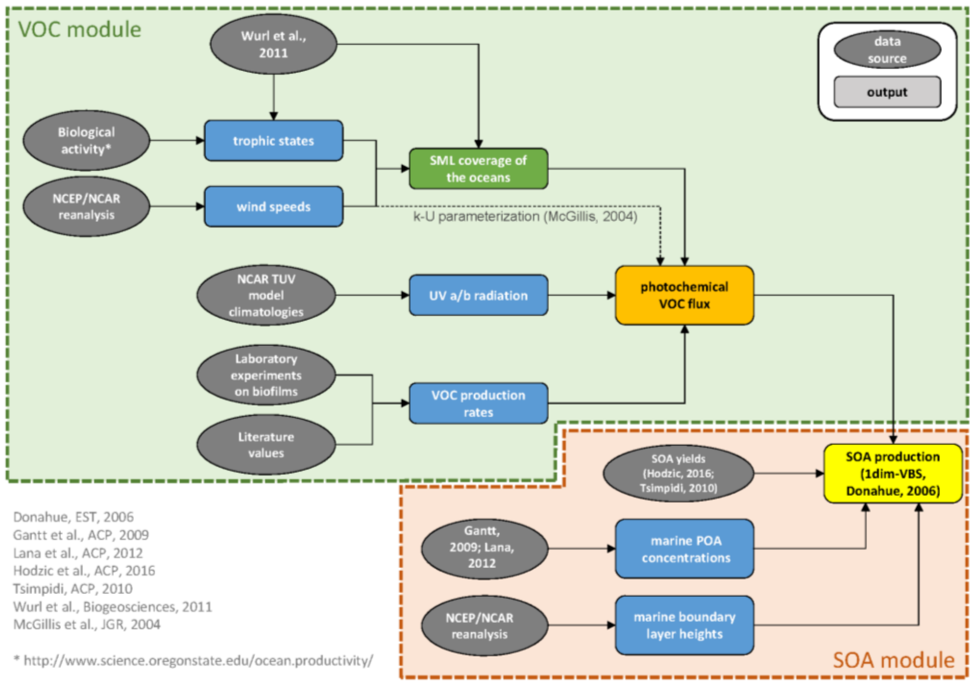

Supplementary Figure 7 | Overview on data resources and calculations conducted using the Photochemistry At Liquid/Air interfaces containing Surfactants (PhotochemAtLAS) model.

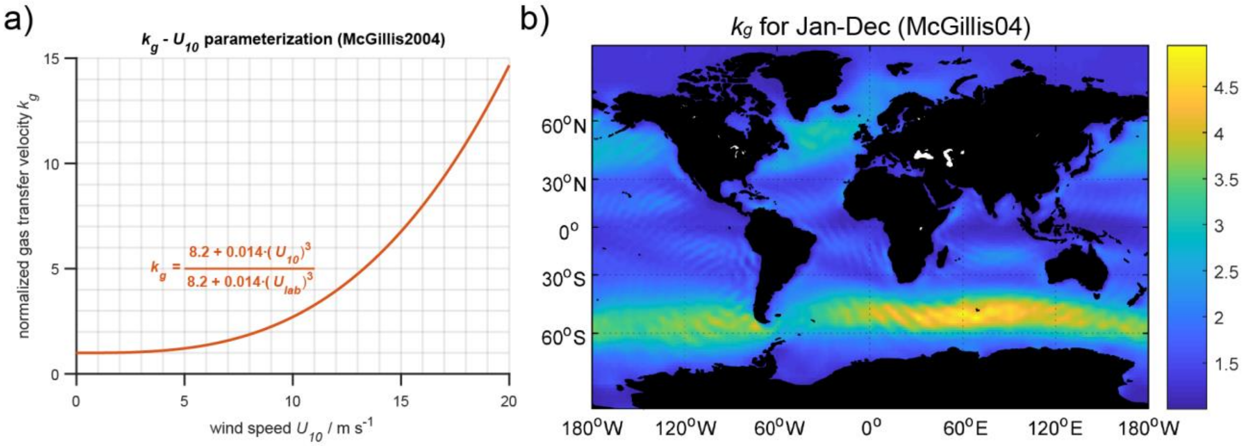

Supplementary Figure 8 | a) Normalized gas transfer velocities k_g, using the k_g–U_{10} parameterization of McGillis *et al.*[5] **b)** Annual distribution of normalized gas transfer velocities k_g. The strongest effect (up to a factor of ~4.7) is observed in the Southern Ocean, where elevated wind speeds enhance air–sea gas transfer velocities (see also Supplementary Fig. 12).

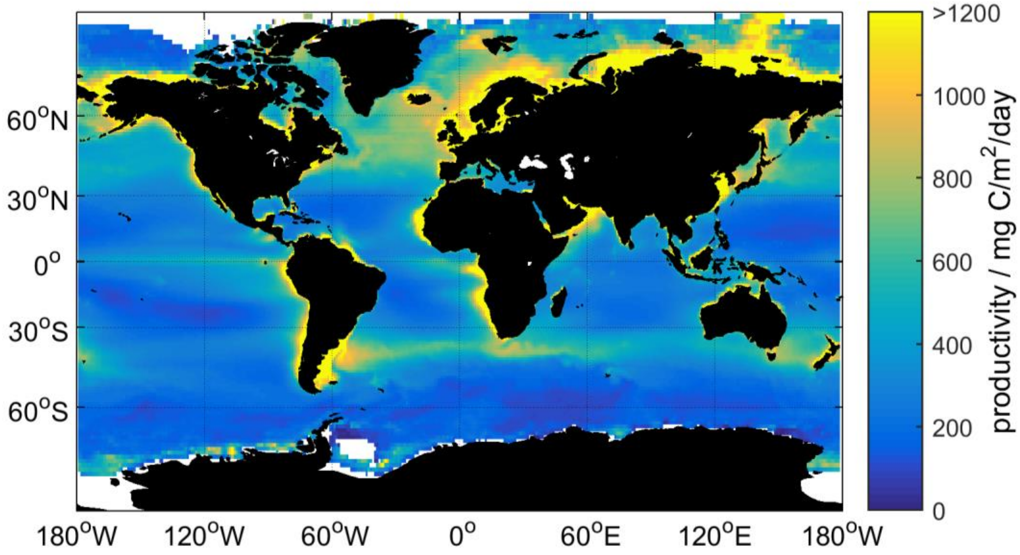

Supplementary Figure 9 | Annual average of net primary productivity, calculated from monthly means from Jul 2002–Nov 2016. Data were obtained from the Ocean Productivity webpage of the Oregon State University (http://www.science.oregonstate.edu/ocean.productivity).

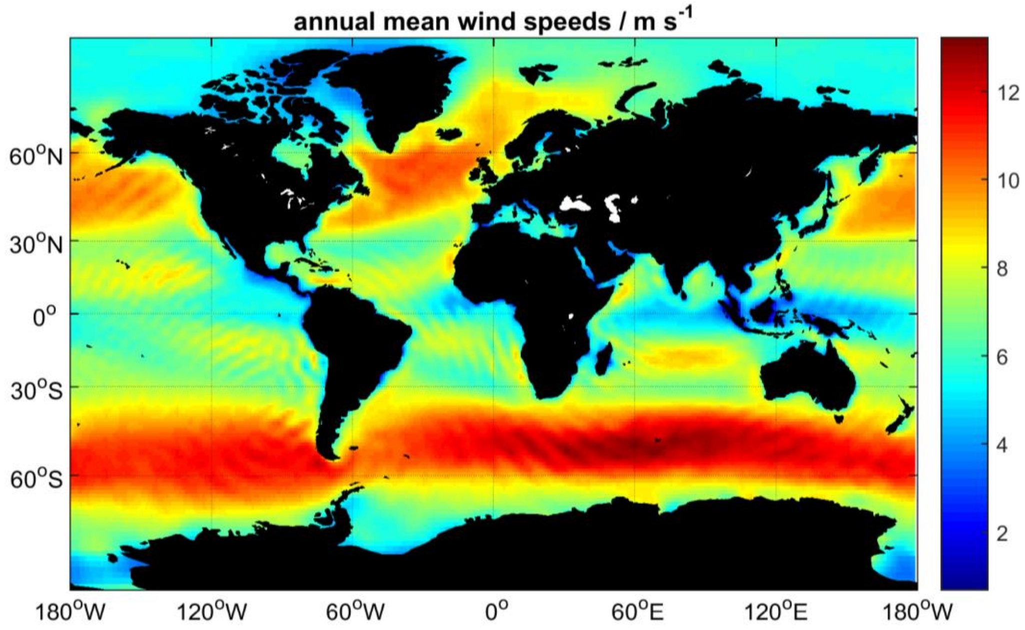

Supplementary Figure 10 | Annual mean wind speeds, calculated from monthly mean wind speeds at 10 meters above the surface of the ocean. Data were obtained from the NCEP/NCAR Reanalysis dataset (https://www.esrl.noaa.gov/).

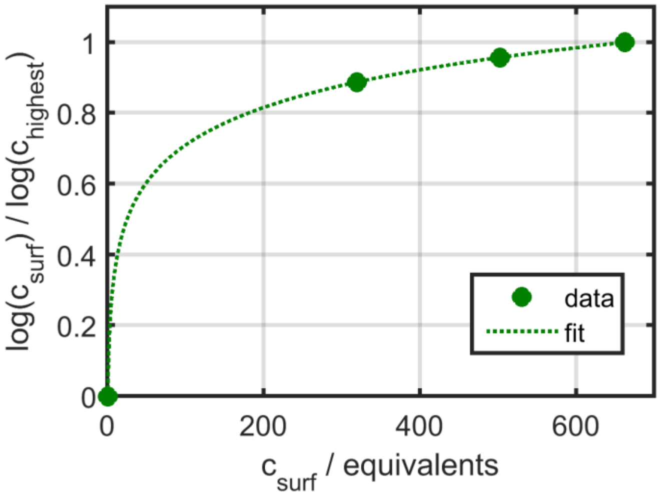

Supplementary Figure 11 | Correction factor as a function of surfactant concentration. According to Ciuraru *et al.* a logarithmic behavior of VOC emissions and surfactant concentration is expected[3,6].

Supplementary Table 2 | List of unsaturated VOCs produced by interfacial photochemistry (identified in laboratory experiments by online-APCI–MS[7]). Reaction rate constants with OH were taken from Atkinson and Arey[8]. For compounds with unknown k_{OH} a chemically similar proxy compound was selected.

formula	photoproduction[a] / 10^7 molecules mW^{-1} s^{-1}	calculated global emission flux / Gg yr^{-1}	compound assignment	k_{OH} / 10^{12} cm^3 molec.$^{-1}$ s^{-1}	proxy compound
C_4H_8	2.659–4.058	477.8–875.5	butene	31.4	
C_5H_6	0.229–0.291	41.15–62.87	cyclopentadiene	35.5	1,2-pentadiene
C_5H_8	3.710–4.788	666.6–1032	isoprene	100	
C_4H_6O	1.655–2.397	297.3–517.0	methacrolein	29	
C_6H_6	0.078–0.092	14.10–19.93	benzene	1.22	
C_6H_8	0.205–0.355	36.77–76.57	cyclohexadiene	164	1,3-cyclohexadiene
C_6H_{10}	1.693–2.317	304.2–499.9	dimethylbutadiene	122	
C_5H_8O	1.087–1.575	195.3–339.7	methylbutenal	86.9	2-methyl-2-butene
C_7H_8	0.050–0.116	9.053–25.09	toluene	5.63	
C_7H_{10}	0.354–0.532	63.66–114.9	dihydrotoluene	164	1,3-cyclohexadiene
C_6H_8O	0.091–0.092	16.42–19.85	hexadienal	112	trans-1,3-hexadiene
C_7H_{12}	0.701–1.023	125.9–220.6	methylcyclohexene	94	
$C_6H_{10}O$	0.273–0.405	48.97–87.29	hexenal	37	1-hexene
C_7H_6O	0.382–0.514	68.66–110.9	benzaldehyde	12	
C_8H_{10}	0.134–0.149	24.15–32.15	xylene (dimethylbenzene)	23.1	
C_8H_{12}	0.397–0.546	71.37–117.8	vinylcyclohexene	94	methylcyclohexene
$C_7H_{10}O$	0.226–0.445	40.67–95.95	cyclohexencarbaldehyde	94	methylcyclohexene
C_8H_{14}	0.534–1.010	96.03–217.8	octadiene	210	2,5-dimethyl-2,4-hexadiene
$C_7H_{12}O$	0.480–0.796	86.23–171.8	heptenal	40	1-heptene
C_9H_{14}	0.264–0.311	47.48–67.00	trimethylcyclohexadiene	164	1,3-cyclohexadiene
$C_8H_{12}O$	0.197–0.218	35.45–47.13	octadienal	210	2,5-dimethyl-2,4-hexadiene
$C_8H_{14}O$	0.812–1.178	145.9–254.1	octenal	69	trans-4-octene
$C_9H_{12}O$	0.039–0.017	7.086–3.573	phenylpropanol	5.8	propanol
$C_9H_{14}O$	0.301–0.332	54.00–71.65	nonadienal	210	2,5-dimethyl-2,4-hexadiene
$C_8H_{12}O_2$	0.029–0.067	5.155–14.44	methylen-2,6-heptandion	26	2,6-dimethyl-4-heptanone
$C_9H_{16}O$	0.401–0.359	71.97–77.44	nonenal	40	1-heptene
$C_{10}H_{14}O$	0.051–0.070	9.137–15.13	carvone	164	limonene
$C_{10}H_{16}O$	0.258–0.281	46.38–60.52	dimethyloctadienal	210	2,5-dimethyl-2,4-hexadiene
**total**	**17.29–24.33**	**3107–5249**	**average**	**109**	

[a] as reported in Brüggemann/Hayeck *et al.*, 2017

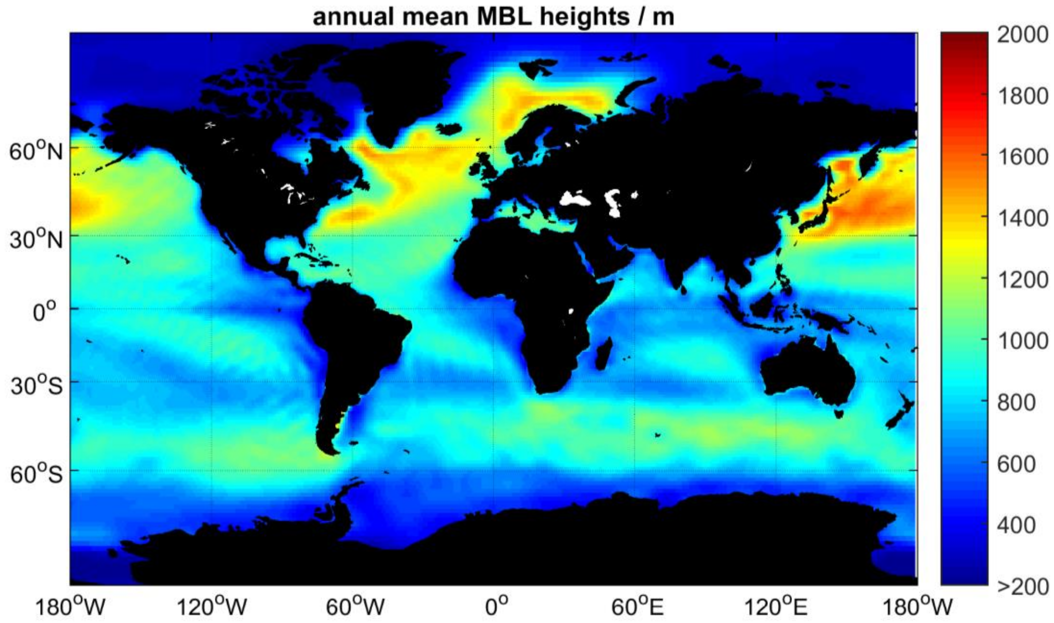

Supplementary Figure 12 | Annual mean MBL heights. Data were obtained from the NOAA-CIRES 20th Century Reanalysis project (https://www.esrl.noaa.gov/psd/data/20thC_Rean/) for the period 1981–2010.

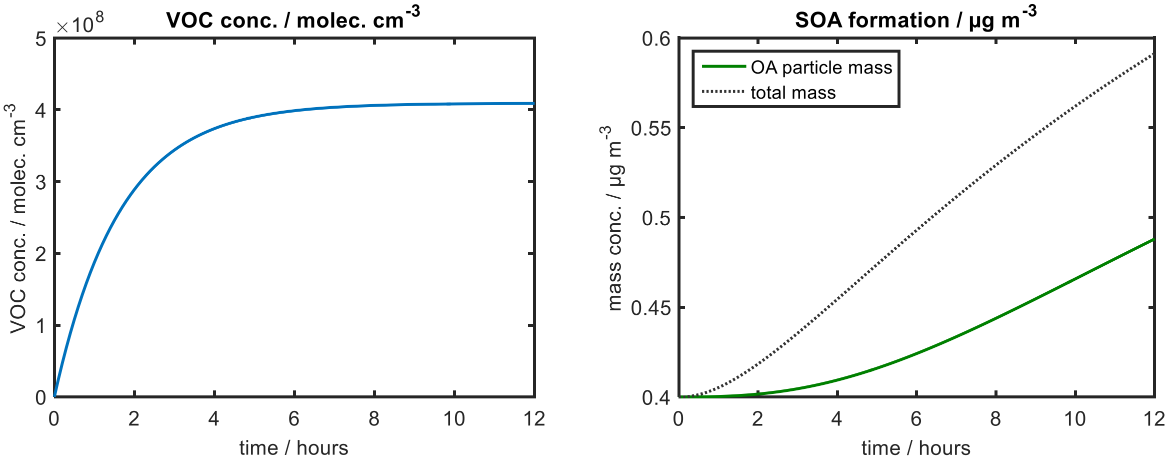

Supplementary Figure 13 | SOA formation within one grid cell from oxidation of VOCs produced from interfacial photochemistry. Photochemistry and OH oxidation are started in parallel at time 0. The left panel shows the mixing ratio of yet unreacted unsaturated VOCs from interfacial photochemistry. The right panel shows the increase in OA mass concentration and the total mass of oxidation products (i.e., gas and particle phase combined).

Supplementary References

1. Lana, A., Simó, R., Vallina, S. M. & Dachs, J. Potential for a biogenic influence on cloud microphysics over the ocean: a correlation study with satellite-derived data. *Atmos. Chem. Phys.* **12,** 7977–7993 (2012).
2. Gantt, B. *et al.* Model evaluation of marine primary organic aerosol emission schemes. *Atmos. Chem. Phys.* **12,** 8553–8566 (2012).
3. Ciuraru, R. *et al.* Photosensitized production of functionalized and unsaturated organic compounds at the air-sea interface. *Sci. Rep.* **5,** 12741 (2015).
4. Sciare, J. *et al.* Long-term observations of carbonaceous aerosols in the Austral Ocean atmosphere: Evidence of a biogenic marine organic source. *J. Geophys. Res.* **114,** D15302 (2009).
5. McGillis, W. R. *et al.* Air-sea CO2 exchange in the equatorial Pacific. *J. Geophys. Res. Ocean.* **109,** C08S2 (2004).
6. Ciuraru, R. *et al.* Unravelling New Processes at Interfaces: Photochemical Isoprene Production at the Sea Surface. *Environ. Sci. Technol.* **49,** 13199–13205 (2015).
7. Brüggemann, M. *et al.* Interfacial photochemistry of biogenic surfactants: a major source of abiotic volatile organic compounds. *Faraday Discuss.* **352,** 357–363 (2017).
8. Atkinson, R. & Arey, J. Atmospheric Degradation of Volatile Organic Compounds. *Chem. Rev.* **103,** 4605–4638 (2003).
